# Long-Term Tea Consumption Is Associated with Reduced Risk of Diabetic Retinopathy: A Cross-Sectional Survey among Elderly Chinese from Rural Communities

**DOI:** 10.1155/2020/1860452

**Published:** 2020-07-12

**Authors:** Cailian Xu, Mingchao Bi, Xuemei Jin, Manhui Zhu, Guohui Wang, Ping Zhao, Xiao Qin, Xun Xu, Xiaodong Sun, Na Ji, Jinxia Du, Jiaowen Xu, Yang Guo, Qinghua Ma, E. Song

**Affiliations:** ^1^Lixiang Eye Hospital of Soochow University, Suzhou, Jiangsu, China; ^2^The First Hospital of Jilin University, Changchun, Jilin, China; ^3^Shanghai Key Laboratory of Ocular Fundus Disease, Shanghai, China; ^4^Department of Ophthalmology, Shanghai First People's Hospital, School of Medicine, Shanghai JiaoTong University, Shanghai, China; ^5^Suzhou Optometry Hospital, Suzhou, Jiangsu, China; ^6^The Third People's Hospital of Xiangcheng District, Suzhou, Jiangsu, China

## Abstract

**Aim:**

To investigate the association between variables related to tea consumption (duration, frequency, and type) and the risk of diabetic retinopathy.

**Methods:**

A rural community-based, cross-sectional survey was conducted in Weitang Town, Suzhou, China. People aged 60 years or above were invited to complete the survey. All eligible patients underwent detailed eye examination. Diabetic retinopathy (DR) was diagnosed and graded based on the retinal fundus imaging. Diabetes was defined as fasting glucose concentrations of ≥7.0 mmol/L or self-reported diagnosis of diabetes. Information about tea consumption such as duration, type, and frequency, together with demographics and lifestyle characteristics, were collected using a face-to-face questionnaire interview. The association between tea consumption and the risk of DR was determined by univariate and multivariate logistic regression analyses.

**Results:**

Among the 5,281 participants, 614 had diabetes mellitus (prevalence of 11.63%). The prevalence rate of DR was 10.38% in the diabetic population and 1.04% in the general population. Compared with non-tea consumers, the crude OR values for DR in subjects with long-term and short-term tea consumption were 0.34 (95%CI = 0.14‐0.82, *p* = 0.016) and 1.64 (95%CI = 0.74‐3.64, *p* = 0.221), respectively. When adjusted for age, gender, and other confounders, consumption of tea for ≥20 years was associated with reduced odds of DR (OR = 0.29, 95%CI = 0.09‐0.97, *p* = 0.044). Thus, long-term tea consumption was significantly associated with a lower risk of DR. There was no statistical significance between frequency or type of tea consumption with DR (*p* > 0.05).

**Conclusion:**

Elderly diabetic Chinese residents who consumed tea for more than twenty years had a lower risk of DR compared to non-tea consumers. The long-term tea consumption may be an independent protective factor for DR. However, further studies are warranted to examine the association.

## 1. Introduction

Diabetic retinopathy (DR) is a chronic, progressive, vision-threatening disease of the retinal microvasculature associated with diabetes mellitus (DM). DR affects approximately one-third of diabetic patients; among these, one-third develop sight-threatening DR [1]. In China, the prevalence of DR has been estimated to be 18.45% in the diabetic population and 1.14% in the general population [2]. DR remains the leading cause of blindness in working-age adults [3], imposing a heavy burden on society [4–9]. The therapeutic methods currently available to treat DR are very limited. In addition, these methods are costly and have relatively poor efficacy [10].

Tea is one of the most consumed beverages in the world. Evidence of tea drinking, especially green tea in China, dates back to the Tang Dynasty. Previous studies have shown that tea consumption is beneficial to human health and may significantly reduce the risk of diabetes and its complications [11]. An earlier meta-analysis suggested that daily tea consumption is associated with a lower risk of DM [12]. Meanwhile, it was found that dietary supplementation of tea products may be helpful in the prevention and treatment of DM [13]. Clinical trials [14, 15] have shown that tea consumption may improve insulin resistance and decrease levels of blood glucose, blood lipid, and blood pressure.

Though tea has shown to possess beneficial effects on diabetes, its role in DR has not yet been clearly identified. Epidemiologic evidence in humans supporting the correlations between tea consumption and DR is even more limited. It has been previously found that diabetic patients regularly drinking green tea had a DR risk reduction of about 50% compared with those who did not drink tea [16]. However, the detailed association between duration, type, or frequency of tea consumption and DR remains unclear. In this study, we hypothesized that tea potentially has a time-cumulative effect on DR, considering that the same phenomenon was observed between tea and DM [17]. In addition, other types of tea might have an impact on DR. Thus, the aim of this study was to investigate the detailed association between variables related to tea consumption (duration, frequency, and type) and DR.

## 2. Materials and Methods

### 2.1. Study Population

This community-based, cross-sectional survey was conducted in Weitang Town, located in Suzhou City, which is famous for its production and culture of Biluochun green tea. These data come from the Weitang Geriatric Diseases Study. Details regarding the methods of this study have previously been described [18, 19]. The study is aimed at estimating the patterns, predictors, and burden of common health outcomes among elderly in eastern China. In brief, a clustering method was used to invite all the elderly residents in the town to participate in the study. Before the study, an invitation letter explaining the nature of the survey was sent to each family. Based on the official records, there were 6,030 elderly people living in Weitang Town. The inclusion criteria are as follows: (1) aged 60 years and above and (2) living for more than six months in Weitang Town by the time the study started. The qualified participants attended the clinic for ocular examination and completed a face-to-face interview. The investigation was carried out from August 2014 to February 2015. The exclusion criteria are as follows: (1) incomplete information on tea consumption and (2) diabetic residents who did not have fundus photographs or whose fundus photographs could not be classified in both eyes.

The study was conducted in conformity with the tenets of the Helsinki Declaration and approved by the Institutional Review Board of Soochow University. All participants gave written informed consents at the recruitment.

### 2.2. Diabetic Retinopathy Assessments

All patients underwent two retinal fundus imaging on both eyes (centered at the optic disk and the macula, respectively) using a digital retinal camera (Canon Inc., Japan). Retinopathy lesions were independently graded by two members of the staff based on the fundus photographs; the retinal specialist was invited to solve an eventual intergrader disagreement. DR was diagnosed and graded according to the Early Treatment of Diabetic Retinopathy Study (ETDRS) criteria [20], a widely used protocol in epidemiologic studies. Patients were divided into two groups: the nondiabetic retinopathy group (non-DR, levels 10–13) and the DR group (levels 14–85). Eyes with severe refractive interstitial opacity, such as a cataract, were excluded because accurate grading of retinal fundus photographs was not possible. Both eyes were evaluated, and the level of retinopathy was based on the eye with the more serious stage of DR if inconsistency occurred in both eyes. The grading was independently performed by two retinal ophthalmologists (CLX and MCB); inconsistency was solved by a senior retinal specialist (ES).

### 2.3. Measurements of Tea Consumptions

The survey collected detailed information on tea consumption using a questionnaire interview. The first question was “Do you usually drink tea?” Subjects who answered “yes” were defined as tea consumers and were asked to choose from or answer the following items: tea type (green tea, black tea, oolong tea, or other types of tea), frequency of tea drinking over the past 12 months (≤1 time/week, 2-3 times/week, 4-5 times/week, and 6-7 times/week), and duration of tea consumption (years). A total of 176 participants who were consuming tea no more than 5 times per week were included in the group “1-5 times/week.” Tea type was classified into green tea and non-green tea since most of the tea drinkers consumed green tea (89.3%). In addition, the tea type was defined as the most regular type of tea which subjects drank, when people consumed multiple teas at the same time. The duration of tea consumption was divided into three groups: non-tea consumption, short-term tea consumption (1-19 years), and long-term tea consumption (≥20 years).

### 2.4. Measurements and Definitions of Other Covariates

All participants underwent a detailed interview using a predesigned questionnaire. Information regarding socioeconomic status (e.g., education), lifestyle risk factors (e.g., smoking and alcohol drinking), medication intake, and history of diseases (e.g., duration of diabetes) was collected. Body height and weight were measured, and the body mass index (BMI) was calculated. The blood pressure (BP) was assessed with the participants sitting for at least 5 minutes. Under fasting conditions, blood samples were collected to determine the concentrations of blood glucose, high-density lipoprotein cholesterol (HDL), low-density lipoprotein cholesterol (LDL), triglyceride, total cholesterol, and similar data. Diabetes was defined as fasting glucose concentrations of ≥7.0 mmol/L or self-reported diagnosis of diabetes, as previously reported [18, 21]. Hypertension was diagnosed with a blood pressure of 140/90 mmHg or higher or on drug treatment for hypertension.

### 2.5. Statistical Analysis

The characteristics of participants were compared using the Student *t*-test for continuous variables and the Chi-square test for categorical variables. The results were expressed as mean values ± standard deviation (SD) for continuous variables or as percentage for categorical variables. The frequency of tea consumption was categorized into three subgroups: 0 time/week (non-tea consumption), 1-5 times/week, and >5 times/week. Besides, tea type was classified into green tea and non-green tea (which included black tea, oolong tea, or other types of tea). Three kinds of categorical variables corresponding to the duration of tea consumption were created: 0 (non-tea consumption), 1–19, and ≥20 years. Both univariate and multivariate logistic regression analyses were performed to study the effect of tea-related variables on DR. For multivariate analysis, only age, gender, and factors that were significantly different in the univariate comparison (*p* < 0.05) or factors of scientific importance were retained in the model. Each outcome variable was expressed with an odds ratio (OR) and 95% confidence interval (95% CI). Two-tailed *p* < 0.05 was considered statistically significant. Analyses were performed using the statistical software Statistical Package for Social Science (SPSS ver.18.0; SPSS Inc., Chicago, IL, USA).

## 3. Results

A total of 5,613 subjects enrolled between August 2014 and January 2015 were eligible. Ultimately, 5,281 residents participated in the current study with a response rate of 94.09%. The age of participants ranged from 60 to 93 years, with a mean of 67.90 ± 6.62 years. Among them, 2,526 (47.83%) were males and 2,755 were (52.17%) females. The patients' characteristics are shown in Table 1. A total of 614 patients were diagnosed with diabetes mellitus (prevalence of 11.63%), and 1812 (34.31%) residents reported regular tea consumption. When comparing the diabetes mellitus and non-diabetes mellitus population, there were no significant differences in age, gender, and duration and type and frequency of tea consumption (all *p* > 0.05).

**Figure 1:**
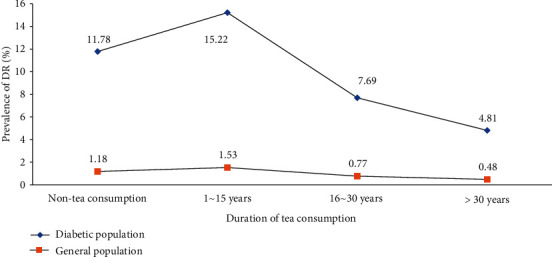
Prevalence of diabetic retinopathy according to the duration of tea consumption.

**Figure 2:**
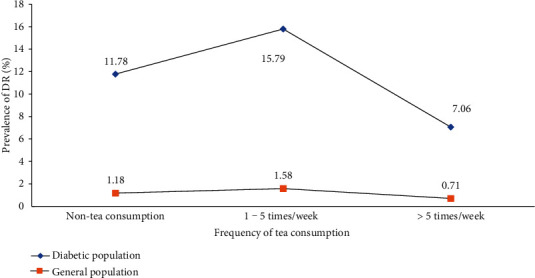
Prevalence of diabetic retinopathy according to the frequency of tea consumption.

**Figure 3:**
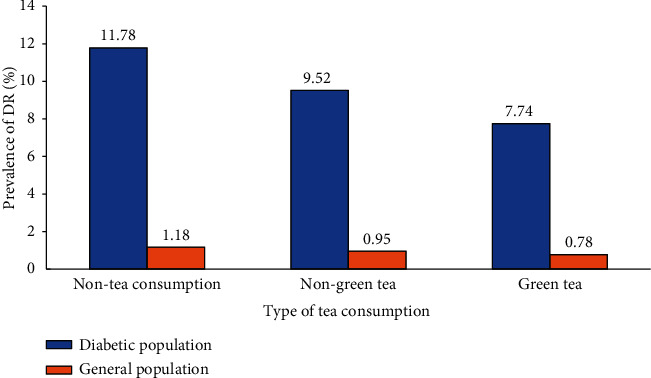
Prevalence of diabetic retinopathy according to the type of tea consumption.

Among 614 diabetic participants, 94 (15.31%) without gradable retinal fundus photographs were mainly excluded due to refractive interstitial opacity such as a cataract. The characteristics of subjects included and excluded from the analysis of tea consumption and diabetic retinopathy are shown in Table S1. Among the 520 diabetic subjects, 241 (46.35%) were men and 279 (53.65%) were women. The mean age of these subjects was 67.05 ± 5.99 (SD) years.  Figure 1 shows the prevalence of DR according to the duration of tea consumption distributed across four groups. The total prevalence rate of DR was 10.38% in the diabetic population and 1.04% in the general population. In detail, this rate among the diabetic population was 11.78% among non-tea consumers, 15.22% for “1~15 years,” and 7.69% for “16~30 years,” 4.81% for “>30 years.” Meanwhile, it was 1.18%, 1.53%, 0.77%, and 0.48% in the general population, respectively. Compared with non-tea drinkers, the prevalence of DR was higher in the short-term tea consumption group and lower in the long-term tea consumption group. Thus, it appeared to be an “inverted U-shaped” correlation between duration of tea consumption and DR. The trend for frequency of tea consumption seemed to be similar (Figure 2). In addition, the prevalence of DR among green-tea consumers was 7.74%, which was lower than 9.52% among non-green tea consumers (Figure 3).

Table 2 shows the univariate logistic regression analysis of the risk factors associated with DR. It was found that the duration of tea consumption was significantly associated with the presence of DR among elderly diabetic patients (odds ratio (OR) = 0.97; per year increase; *p* = 0.02). Compared with non-tea consumers, the crude OR values for DR in subjects with long-term and short-term tea consumption were 0.34 (95%CI = 0.14‐0.82, *p* = 0.016) and 1.64 (95%CI = 0.74‐3.64, *p* = 0.221), respectively. Thus, long-term tea consumption was significantly associated with a lower risk of DR. Yet, the difference between short-term tea consumption and non-tea consumption associated with DR was insignificant (*p* > 0.05). Similarly, the risk of DR was likely to increase first and then decrease with the frequency of tea consumption, yet insignificantly. Moreover, no statistically significant associations were found either in relation to the type of tea consumption or in relation to other variables, such as age, gender, or serological variables with DR (*p* > 0.05). After adjusting for multiple factors, the trend of correlation between the duration of tea consumption and DR was still consistent with univariate analysis (Table 3). Compared with those who did not drink tea, the adjusted OR for DR in long-term tea consumers was 0.29 (95%CI = 0.09‐0.97, *p* = 0.044). Thus, the long-term duration of tea consumption was possibly an independent factor of DR in elderly subjects with diabetes.

## 4. Discussion

The current study initially reported on the association between duration of tea consumption and diabetic retinopathy in a community-based cross-sectional survey among the elderly Chinese population. This study suggested that long-term tea consumption is significantly associated with a lower risk of DR. Among Chinese diabetic subjects aged 60 years or older, tea consumers had a reduced DR risk by 3% per year for an increasing duration of tea consumption. When adjusting for various confounding factors, those who consumed tea for more than twenty years had a significantly decreased risk for DR of 71% compared to non-tea drinkers, while those who consumed tea for 1-19 years had an insignificantly increased risk of 64%. In addition, frequency and type of tea consumption were also insignificantly associated with DR. Therefore, our data suggested that long-term tea consumption had an independent protective effect on DR. But future, longitudinal studies are needed to test this hypothesis.

Previous reports have shown that tea consumption might affect human health in a dose-dependent manner [22]. Yet, according to our knowledge, few studies reported on the association between tea and DR [23], including our own studies [16, 24]. There is especially a lack of studies focusing directly on the correlation between duration of tea consumption and DR. In the present work, the prevalence of DR was observed to likely increase first and then to decrease with the duration of tea consumption. Thus, there might be a similar “inverted U-shaped” relationship between the duration of tea consumption and DR. However, compared with non-tea consumers, the increased risk of DR among short-term tea consumers was not statistically significant after logistic regression analysis; only long-term tea consumption (twenty years above) revealed a positive association with a lower risk of DR. Combining the result that tea consumers tended to have a DR risk reduction of 3% per year for an increasing duration of tea consumption, it is suggested that the protective impact of tea on DR is probably by accumulation of time, and in other words, maybe only long-term tea consumption has a beneficial effect on DR compared to short-term tea consumption.

It has been reported that frequent tea consumption for more than ten years could reduce the percent of body fat and waist-to-hip ratio by 19.6% and 2.1% [25]. Moreover, a Japanese study revealed that those who consumed green tea everyday for five years had a 42% lower risk of diabetes [26]. Tea or tea extract was reported to increase the antioxidant capacity of serum in diabetes [27, 28], exert neuroprotective properties in DR [29], inhibit ocular neovascularization and vascular permeability [30], reduce systolic blood pressure and enhance both endothelial function and insulin sensitivity [31], and slow down age-related decreases in HDL-C concentrations [32]. Over all, tea consumption could block almost all the factors influencing the development of DR. It is generally known that the duration of diabetes is the leading risk factor of DR [33]. For long-term tea consumers with DR, the effect of tea can intervene in early diabetes, even before the onset of the disease. Moreover, we found that frequency and type of tea consumption were not significantly associated with the risk of DR. This might be due to the small sample size in some categories, since the numbers of participants with DR drinking tea for 1–5 times/week or drinking non-green tea were 3 (5.56%) and 2 (3.70%), respectively. Therefore, the exact correlation between frequency and type of consumed tea in those with DR still remains uncertain, requiring further validation of these findings.

Besides, the data indicated that the prevalence of DR was 10.38% in the diabetic population and 1.04% in the general population. Partial population-based studies conducted in the Chinese population are shown in Table 4. The prevalence of DR in the current study was higher than the Yangxi Eye Study, with a prevalence of 8.2% [34]. In our study, the ratio between known diabetes mellitus to newly diabetes mellitus was 3 : 1, which was larger than the 1 : 5 reported in the Yangxi Eye Study. The lower prevalence of DR may be explained by the shorter duration of diabetes. Nonetheless, our observed prevalence of any DR was lower than those reported in most of other studies with values ranging from 18.0% to 43.1% [35–39]. A meta-analysis reported a prevalence of 18.5% in the Chinese population between 1990 and 2017 [2]. This survey was conducted in Suzhou, famous for its production and culture of Biluochun green tea. The lower prevalence of DR in the current population may be attributed to the fact that local older adults habitually drink tea. In order to validate the assumption, more research on this topic needs to be done in other tea-drinking areas.

The present study has a few limitations. Firstly, diabetes was determined based on fasting blood glucose, referring to the Beijing Eye Study [38] and the Handan Eye Study [39]. However, oral glucose tolerance test and HbA1c were not performed in the study. Because our results were obtained from subgroup analysis of the Weitang Geriatric Diseases Study [18, 19], and because the primary goal of the total project was not focused on diabetes initially, subjects with prediabetes were not included in the tea-related analysis. Also, multivariate logistic regression analysis models of tea consumption and DR could not be adjusted for HbA1c. Still, we have adjusted FBG, and self-reported glycemic control was represented instead in an indirect way. Secondly, comprehensive dietary intake information, such as intake of fruits, vegetables, and meat, were missing. Yet, in the analysis, we controlled some closely diet-related factors, including BMI and alcohol intake, which can reduce the confounding to some extent. Thirdly, there was potential bias due to those excluded from the analysis being older and having a longer duration of diabetes, because these variables are recognized as the main factors influencing DR [2, 40]. Thus, the prevalence of DR might have been less well assessed in the present study. Fourthly, information on tea consumption was collected by interview questionnaire. This approach is known to recall biases, especially among people aged 60 years and above. Finally, as the current study was cross-sectional, it was difficult to infer the causal relationship between tea consumption and DR. Hence, reported observations need to be further confirmed in well-designed cohort studies, including studies with a larger diabetic population.

This study also has several strengths. Subjects were recruited from the community rather than the clinic. Besides, the response rate of 94.09% in the study was relatively high, which could reduce the possibility of selection bias and enable a more comprehensive evaluation of the research results. Moreover, to the best of our knowledge, this is the first epidemiological study that investigated a possible association between the duration of tea consumption and DR prevalence in the Chinese population. Our data indicated the potential effect of tea consumption against DR. Tea is regarded as a potential hypoglycemic substance with low cost, good patient compliance, and fewer side effects compared with many synthetic drugs [41]. Thus, tea consumption has good potential to be applied for clinical and public prevention against DR. However, it should be noted that tea consumption might not have an effect on DR until a long time of accumulation. Despite the growing evidence, further studies are needed to confirm how tea consumption should be recommended to the general or high-DR-risk population groups.

## 5. Conclusions

The duration of tea consumption is associated with diabetic retinopathy in the elderly Chinese population. Long-term tea consumption may be a possible independent protective factor for diabetic retinopathy. The correlation between frequency or type of tea consumption with DR still remains uncertain. Thus, longitudinal cohort studies with a larger diabetic population are warranted to confirm these results.

## Figures and Tables

**Table 1:** Characteristics of the study population with and without diabetes mellitus.

	Total (*n* = 5,281)		DM (*n* = 614)		Non-DM (*n* = 4,667)		*p*
Age (y)	67.90 ± 6.62		68.03 ± 6.49		67.88 ± 6.64		0.584
Gender							
Male	2,526	47.83%	279	45.44%	2,247	48.15%	
Female	2,755	52.17%	335	54.56%	2,420	51.85%	0.207
Occupation							
Peasant	2,956	55.97%	346	56.35%	2,610	55.92%	
Other	2,325	44.03%	268	43.65%	2,057	44.08%	0.841
Educational level							
Illiterate or no education	2,566	48.59%	295	48.05%	2,271	48.66%	
Primary education and above	2,715	51.41%	319	51.95%	2,396	51.34%	0.774
Individual monthly income							
≤¥2000	4,327	81.98%	486	79.15%	3,841	82.35%	
>¥2000	951	18.02%	128	20.85%	823	17.65%	0.052
Smoking							
No	3,917	74.17%	481	78.34%	3,436	73.62%	
Yes	1,364	25.83%	133	21.66%	1,231	26.38%	**0.012**
Alcohol consumption							
No	4,094	77.52%	484	78.83%	3,610	77.35%	
Yes	1,187	22.48%	130	21.17%	1,057	22.65%	0.410
Type of tea consumption							
Non-tea consumption	3,469	65.69%	395	64.33%	3,074	65.87%	
Green tea	1,619	30.66%	196	31.92%	1,423	30.49%	
Non-green tea	193	3.65%	23	3.75%	170	3.64%	0.751
Frequency of tea consumption							
Non-tea consumption	3,469	65.69%	395	64.33%	3,074	65.87%	
1–5 times/week	192	3.64%	20	3.26%	172	3.69%	
>5 times/week	1,620	30.68%	199	32.41%	1,421	30.45%	0.563
Duration of tea consumption (y)	10.25 ± 17.03		10.99 ± 17.73		10.13 ± 16.93		0.253

*Note*. DM: diabetes mellitus; non-DM: non-diabetes mellitus. Bold type indicates statistical significance (< 0.05).

**Table 2:** Univariable logistic regression analysis of the risk factors associated with diabetic retinopathy.

	Total (*n* = 520)	DR (*n* = 54)	Non-DR (*n* = 446)	OR (95% CI)	*p*
	Number (%)				
Gender					
Male	241 (46.35)	19 (35.19)	222 (47.64)	1	Ref
Female	279 (53.65)	35 (64.81)	244 (52.36)	1.68 (0.93–3.02)	0.085
Occupation					
Peasant	285 (54.81)	34 (62.96)	251 (53.86)	1	Ref
Other	235 (45.19)	20 (37.04)	215 (46.14)	0.69 (0.38–1.23)	0.203
Educational level					
Illiterate or no education	239 (45.96)	29 (53.70)	210 (45.06)	1	Ref
Primary education and above	281 (54.04)	25 (46.30)	256 (54.94)	0.71 (0.40–1.24)	0.228
Individual monthly income					
≤¥2,000	406 (78.08)	38 (70.37)	368 (78.97)	1	Ref
>¥2,000	114 (21.92)	16 (29.63)	98 (21.03)	1.58 (0.85–2.95)	0.148
Diabetes diagnosis					
Newly	137 (26.35)	17 (31.48)	120 (25.75)	1	Ref
Previously	383 (73.65)	37 (68.52)	346 (74.25)	0.75 (0.41–1.39)	0.367
Insulin					
No	450 (86.54)	47 (86.48)	403 (86.48)	1	Ref
Yes	70 (13.46)	7 (13.52)	63 (13.52)	0.953 (0.41–2.20)	0.910
Self-reported glycemic control					
Poor	458 (88.08)	52 (96.30)	406 (87.12)	1	Ref
Good	62 (11.92)	2 (3.70)	60 (12.88)	0.26 (0.06–1.10)	0.067
Hypertension					
No	159 (30.58)	14 (25.93)	145 (31.12)	1	Ref
Yes	361 (69.42)	40 (74.07)	321 (68.88)	1.529 (0.70–3.35)	0.289
Smoking					
No	402 (77.31)	44 (81.48)	358 (76.82)	1	Ref
Yes	118 (22.69)	10 (18.52)	108 (23.18)	0.75 (0.37–1.55)	0.44
Alcohol consumption					
No	404 (77.69)	44 (81.48)	360 (77.25)	1	Ref
Yes	116 (22.31)	10 (18.52)	106 (22.75)	0.77 (0.38–1.59)	0.481
Type of tea consumption					
Non-tea consumption	331 (63.65)	39 (72.22)	292 (62.66)	1	Ref
Green tea	168 (32.31)	13 (24.07)	155 (33.26)	0.63 (0.33–1.21)	0.165
Non-green tea	21 (4.04)	2 (3.70)	19 (4.08)	0.79 (0.18–3.51)	0.755
Frequency of tea consumption					
Non-tea consumption	331 (63.65)	39 (72.22)	292 (62.66)	1	Ref
1–5 times/week	19 (3.65)	3 (5.56)	16 (3.43)	1.40 (0.39–5.04)	0.603
>5 times/week	170 (32.69)	12 (22.22)	158 (33.91)	0.57 (0.29–1.12)	0.101
Duration of tea consumption group					
Non-tea consumption	331 (63.65)	39 (72.22)	292 (62.66)	1	Ref
1~19 years	50 (9.62)	9 (16.67)	41 (8.80)	1.64 (0.74–3.64)	0.221
≥20 years	139 (26.73)	6 (11.11)	133 (28.54)	0.34 (0.14–0.82)	**0.016**

	Mean (standard deviation)				
Age (y)	67.05 ± 5.99	67.13 ± 5.61	67.04 ± 6.04	1.00 (0.96–1.05)	0.918
Duration of tea consumption (y)	10.87 ± 17.24	5.54 ± 11.83	11.48 ± 17.67	0.97 (0.95–1.00)	**0.020**
Duration of diabetes (y)	5.41 ± 5.80	6.33 ± 7.80	5.30 ± 5.52	1.03 (0.98–1.07)	0.218
BMI (kg/m^2^)	24.34 ± 3.06	24.74 ± 3.01	24.40 ± 3.00	1.03 (0.92–1.15)	0.584
FBG (mmol/L)	7.72 ± 2.02	8.31 ± 2.82	7.71 ± 1.89	1.12 (0.99–1.26)	0.075
TC (mmol/L)	4.70 ± 0.97	4.60 ± 0.93	4.71 ± 1.00	0.91 (0.67–1.24)	0.540
TG (mmol/L)	1.60 ± 1.08	1.48 ± 0.67	1.65 ± 1.22	0.81 (0.57–1.16)	0.257
HDL-C (mmol/L)	1.34 ± 0.36	1.30 ± 0.29	1.34 ± 0.36	0.64 (0.27–1.55)	0.324
LDL-C (mmol/L)	2.77 ± 0.79	2.77 ± 0.71	2.77 ± 0.82	1.07 (0.74–1.55)	0.727
SBP (mm Hg)	149.46 ± 19.40	152.79 ± 18.81	149.3 ± 18.92	1.01 (1.00–1.03)	0.063
DBP (mm Hg)	86.99 ± 11.09	86.24 ± 11.36	87.14 ± 10.83	1.00 (0.98–1.03)	0.909

*Note*. DR: diabetic retinopathy; non-DR: nondiabetic retinopathy; OR: odds ratio; 95% CI: 95% confident interval; BMI: body mass index; FBG: fasting blood glucose; TC: total cholesterol; TG: triglyceride; HDL-C: high-density lipoprotein cholesterol; LDL-C: low-density lipoprotein cholesterol; SBP: systolic blood pressure; DBP: diastolic blood pressure. Bold type indicates statistical significance (*p* < 0.05).

**Table 3:** Multivariate logistic regression models of tea consumption and diabetic retinopathy.

	Number of		Model 1^a^			Model 2^b^			Model 3^c^		
	DR	Non-DR	B	OR (95% CI)	*p*	B	OR (95% CI)	*p*	B	OR (95% CI)	*p*
Duration of tea consumption (y)	—	—	-0.02	0.98 (0.96–1.00)	0.059	-0.03	0.97 (0.95–1.00)	**0.041**	-0.03	0.97 (0.95–1.00)	**0.046**
Duration of tea consumption group											
Non-tea consumption	39	292	0	1	Ref	0	1	Ref	0	1	Ref
1–19 years	9	41	0.55	1.74 (0.77–3.91)	0.183	0.44	1.55 (0.66–3.65)	0.318	0.57	1.76 (0.65–4.77)	0.263
≥20 years	6	133	-0.95	0.39 (0.15–1.00)	0.051	-0.99	0.37 (0.14–0.97)	**0.042**	-1.22	0.29 (0.09–0.97)	**0.044**
Frequency of tea consumption											
Non-tea consumption	39	292	0	1	Ref	0	1	Ref	0	1	Ref
1–5 times/week	3	16	-0.39	0.68 (0.32–1.45)	0.319	-0.54	0.58 (0.27–1.28)	0.179	-0.7	0.50 (0.19–1.29)	0.15
>5 times/week	12	158	0.37	1.44 (0.40–5.19)	0.576	0.4	1.48 (0.41–5.41)	0.549	0.86	2.37 (0.57–9.84)	0.236
Tea type											
Non-tea consumption	39	292	0	1	Ref	0	1	Ref	0	1	Ref
Green tea	13	155	-0.26	0.77 (0.37–1.61)	0.487	-0.39	0.68 (0.32–1.45)	0.315	-0.31	0.74 (0.30–1.79)	0.5
Non-green tea	2	19	-0.15	0.86 (0.19–3.88)	0.847	-0.19	0.83 (0.18–3.76)	0.806	-0.79	0.46 (0.05–4.01)	0.479

*Note*. DR: diabetic retinopathy; OR: odds ratio; 95% CI: 95% confident interval. ^a^Model 1 was adjusted for age and gender. ^b^Model 2 was adjusted for age, gender, and covariates with *p* < 0.2 in univariate analysis in Table 3, including individual monthly income, FBG, and SBP. ^c^Model 3 was adjusted for age, gender, and covariates with *p* < 0.5 in univariate analysis in Table 3, including occupation, educational level, individual monthly income, smoking, alcohol consumption, duration of diabetes, BMI, FBG, TG, HDL-C, and SBP. Bold type indicates statistical significance (*p* < 0.05).

**Table 4:** Prevalence of DR in partial population-based studies for the Chinese race.

Study	Date of data collection	Setting	Population	Mean age (y)	Sample	Prevalence of DR^#^
This study	2014–2015	Rural	General	67.9 ± 6.6	5,281	10.4%
Yangxi Eye Study [34]	2014	Rural	General	65.7 ± 9.5	5,258	8.2%
Gusu DR screening study [35]	2015	Urban	Diabetic	67.7 ± 8.3	913	18.0%
Desheng Diabetic Eye Study [36]	2009–2012	Urban	Diabetic	64.8 ± 8.2	1,340	35.2%
Chinese American Eye Study [37]	2010–2013	Urban	General	≥50	4,582	35.8%
Beijing Eye Study [38]	2006	Urban and rural	General	60.4 ± 10.0	3,251	27.9%
Handan Eye Study [39]	2006–2007	Rural	General	51.7 ± 11.4	6,830	43.1%
Meta-analysis study [2]	2017	Urban and rural	General	NA	NA	18.5%

NA: data was not available. ^#^Prevalence of DR is for the diabetic population.

## Data Availability

The raw/processed data required to reproduce these findings cannot be shared at this time as these data also form part of an ongoing study.

## Supplementary Materials

Supplementary Materials Table S1: comparison of subjects included and excluded from the analysis.

## References

[1] Yau J. W. Y., Rogers S. L., Kawasaki R. (2012). Global prevalence and major risk factors of diabetic retinopathy. *Diabetes Care*.

[2] Song P., Yu J., Chan K. Y., Theodoratou E., Rudan I. (2018). Prevalence, risk factors and burden of diabetic retinopathy in China: a systematic review and meta-analysis. *Journal of Global Health*.

[3] Cheung N., Mitchell P., Wong T. Y. (2010). Diabetic retinopathy. *Lancet*.

[4] Cikamatana L., Mitchell P., Rochtchina E., Foran S., Wang J. J. (2007). Five-year incidence and progression of diabetic retinopathy in a defined older population: the Blue Mountains Eye Study. *Eye*.

[5] Clark A., Morgan W. H., Kain S. (2010). Diabetic retinopathy and the major causes of vision loss in Aboriginals from remote Western Australia. *Clinical & Experimental Ophthalmology*.

[6] Kempen J. H., O'Colmain B. J., Leske M. C. (2004). The prevalence of diabetic retinopathy among adults in the United States. *Archives of Ophthalmology*.

[7] McKay R., McCarty C. A., Taylor H. R. (2000). Diabetic retinopathy in Victoria, Australia: the Visual Impairment Project. *The British Journal of Ophthalmology*.

[8] Wong T. Y., Klein R., Islam F. M. A. (2006). Diabetic retinopathy in a multi-ethnic cohort in the United States. *American Journal of Ophthalmology*.

[9] Xu J., Wei W. B., Yuan M. X. (2012). Prevalence and risk factors for diabetic retinopathy: the Beijing Communities Diabetes Study 6. *Retina*.

[10] Lin J., Chang J. S., Smiddy W. E. (2016). Cost evaluation of panretinal photocoagulation versus intravitreal ranibizumab for proliferative diabetic retinopathy. *Ophthalmology*.

[11] Meng J.-M., Cao S.-Y., Wei X.-L. (2019). Effects and mechanisms of tea for the prevention and management of diabetes mellitus and diabetic complications: an updated review. *Antioxidants*.

[12] Yang J., Mao Q. X., Xu H. X., Ma X., Zeng C. Y. (2014). Tea consumption and risk of type 2 diabetes mellitus: a systematic review and meta-analysis update. *BMJ Open*.

[13] Moon H. S., Lee H. G., Choi Y. J., Kim T. G., Cho C. S. (2007). Proposed mechanisms of (-)-epigallocatechin-3-gallate for anti-obesity. *Chemico-Biological Interactions*.

[14] Mousavi A., Vafa M., Neyestani T., Khamseh M., Hoseini F. (2013). The effects of green tea consumption on metabolic and anthropometric indices in patients with type 2 diabetes. *Journal of Research in Medical Sciences : The Official Journal of Isfahan University of Medical Sciences*.

[15] Liu C. Y., Huang C. J., Huang L. H., Chen I. J., Chiu J. P., Hsu C. H. (2014). Effects of green tea extract on insulin resistance and glucagon-like peptide 1 in patients with type 2 diabetes and lipid abnormalities: a randomized, double-blinded, and placebo-controlled trial. *PLoS One*.

[16] Ma Q., Chen D., Sun H.-P., Yan N., Xu Y., Pan C.-W. (2015). Regular Chinese green tea consumption is protective for diabetic retinopathy: a clinic-based case-control study. *Journal of Diabetes Research*.

[17] Nguyen C. T., Lee A. H., Pham N. M. (2018). Habitual tea drinking associated with a lower risk of type 2 diabetes in Vietnamese adults. *Asia Pacific Journal of Clinical Nutrition*.

[18] Pan C.-W., Wang X., Ma Q., Sun H.-P., Xu Y., Wang P. (2015). Cognitive dysfunction and health-related quality of life among older Chinese. *Scientific Reports*.

[19] Xu C., Pan C., Zhao C. (2017). Prevalence and risk factors for myopia in older adult east Chinese population. *BMC Ophthalmology*.

[20] Early Treatment Diabetic Retinopathy Study Research Group (1991). Grading Diabetic Retinopathy from Stereoscopic Color Fundus Photographs—An Extension of the Modified Airlie House Classification: ETDRS Report Number 10. *Ophthalmology*.

[21] Xie X. W., Xu L., Wang Y. X., Jonas J. B. (2008). Prevalence and associated factors of diabetic retinopathy. The Beijing Eye Study 2006. *Graefe's Archive for Clinical and Experimental Ophthalmology*.

[22] Saito E., Inoue M., Sawada N. (2015). Association of green tea consumption with mortality due to all causes and major causes of death in a Japanese population: the Japan Public Health Center-based Prospective Study (JPHC Study). *Annals of Epidemiology*.

[23] Dow C., Mancini F., Rajaobelina K. (2018). Diet and risk of diabetic retinopathy: a systematic review. *European Journal of Epidemiology*.

[24] Wang L., Sun X., Zhu M. (2019). Epigallocatechin-3-gallate stimulates autophagy and reduces apoptosis levels in retinal Müller cells under high-glucose conditions. *Experimental Cell Research*.

[25] Wu C. H., Lu F. H., Chang C. S., Chang T. C., Wang R. H., Chang C. J. (2003). Relationship among habitual tea consumption, percent body fat, and body fat distribution. *Obesity Research*.

[26] Iso H., Date C., Wakai K., Fukui M., Tamakoshi A., and the JACC Study Group (2006). The relationship between green tea and total caffeine intake and risk for self-reported type 2 diabetes among Japanese adults. *Annals of Internal Medicine*.

[27] Toolsee N. A., Aruoma O. I., Gunness T. K. (2013). Effectiveness of green tea in a randomized human cohort: relevance to diabetes and its complications. *BioMed Research International*.

[28] Kumar B., Gupta S. K., Nag T. C., Srivastava S., Saxena R. (2012). Green tea prevents hyperglycemia-induced retinal oxidative stress and inflammation in streptozotocin-induced diabetic rats. *Ophthalmic Research*.

[29] Silva K. C., Rosales M. A. B., Hamassaki D. E. (2013). Green tea is neuroprotective in diabetic retinopathy. *Investigative Ophthalmology & Visual Science*.

[30] Lee H., Jun J.-H., Jung E.-H., Koo B., Kim Y. (2014). Epigalloccatechin-3-gallate inhibits ocular neovascularization and vascular permeability in human retinal pigment epithelial and human retinal microvascular endothelial cells via suppression of MMP-9 and VEGF activation. *Molecules*.

[31] Potenza M. A., Marasciulo F. L., Tarquinio M. (2007). EGCG, a green tea polyphenol, improves endothelial function and insulin sensitivity, reduces blood pressure, and protects against myocardial I/R injury in SHR. *American Journal of Physiology. Endocrinology and Metabolism*.

[32] Huang S., Li J., Wu Y. (2018). Tea consumption and longitudinal change in high-density lipoprotein cholesterol concentration in Chinese adults. *Journal of the American Heart Association*.

[33] Simo-Servat O., Hernandez C., Simo R. (2019). Diabetic retinopathy in the context of patients with diabetes. *Ophthalmic Research*.

[34] Jin G., Xiao W., Ding X. (2018). Prevalence of and risk factors for diabetic retinopathy in a rural Chinese population: the Yangxi Eye Study. *Investigative Ophthalmology & Visual Science*.

[35] Pan C. W., Wang S., Qian D. J., Xu C., Song E. (2017). Prevalence, awareness, and risk factors of diabetic retinopathy among adults with known type 2 diabetes mellitus in an urban community in China. *Ophthalmic Epidemiology*.

[36] Yang X., Deng Y., Gu H. (2016). Relationship of retinal vascular calibre and diabetic retinopathy in Chinese patients with type 2 diabetes mellitus: the Desheng Diabetic Eye Study. *The British Journal of Ophthalmology*.

[37] Varma R., Wen G., Jiang X. (2016). Prevalence of diabetic retinopathy in adult Chinese American individuals: the Chinese American Eye Study. *JAMA Ophthalmology*.

[38] Xie X. W., Xu L., Jonas J. B., Wang Y. X. (2018). Prevalence of diabetic retinopathy among subjects with known diabetes in China: the Beijing Eye Study. *European Journal of Ophthalmology*.

[39] Wang F. H., Liang Y. B., Zhang F. (2009). Prevalence of diabetic retinopathy in rural China: the Handan Eye Study. *Ophthalmology*.

[40] Voigt M., Schmidt S., Lehmann T. (2018). Prevalence and progression rate of diabetic retinopathy in type 2 diabetes patients in correlation with the duration of diabetes. *Experimental and Clinical Endocrinology & Diabetes*.

[41] Mahmoud F., Haines D., Al-Ozairi E., Dashti A. (2016). Effect of black tea consumption on intracellular cytokines, regulatory T cells and metabolic biomarkers in type 2 diabetes patients. *Phytotherapy Research*.

