# Supplementary material for: Long-Term Tea Consumption Is Associated with Reduced Risk of Diabetic Retinopathy: A Cross-Sectional Survey among Elderly Chinese from Rural Communities
Source: J Diabetes Res. 2020 Jul 12;2020:1860452. doi: 10.1155/2020/1860452 (PMC7372293; doi:10.1155/2020/1860452)
Supplement: Supplementary Materials — Table S1: comparison of subjects included and excluded from the analysis. [file 1860452.f1.pdf]

Supplementary materials

Table S1. Comparison of subjects included and excluded from the analysis

			Excluded		Included		
	N = 614	%	N = 94	%	N = 520	%	*p*
Age, y	68.03 ± 6.48		73.47 ± 6.44		67.05 ± 5.99		**< 0.001**
Gender							
Male	279	45.44	38	40.43	241	46.35	
Female	335	54.56	56	59.57	279	53.65	0.289
Occupation							
Peasant	346	56.35	61	64.89	285	54.81	
Other	268	43.65	33	35.11	235	45.19	0.070
Educational level							
Illiterate or no education	295	48.05	56	59.57	239	45.96	
Primary education and above	319	51.95	38	40.43	281	54.04	**0.015**
Individual monthly income							
⩽ ¥2000	486	79.15	80	85.11	406	78.08	
> ¥2000	128	20.85	14	14.89	114	21.92	0.123
Diabetes diagnosis							
Newly	173	28.18	36	38.30	137	26.35	
Previously	441	71.82	58	61.70	383	73.65	**0.018**
Duration of diabetes, y	5.64 ± 6.24		6.91 ± 8.18		5.41 ± 5.80		**0.031**
Smoking							
No	481	78.34	79	84.04	402	77.31	
Yes	133	21.66	15	15.96	118	22.69	0.145
Alcohol consumption							

No	484	78.83	80	85.11	404	77.69	
Yes	130	21.17	14	14.89	116	22.31	0.105
Type of tea consumption							
Non-tea consumption	395	64.33	64	68.09	331	63.65	
Green tea	196	31.92	28	29.79	168	32.31	
Other	23	3.75	2	2.13	21	4.04	0.313
Frequency of tea consumption							
Non-tea consumption	395	64.33	64	68.09	331	63.65	
1 – 5 times/week	199	32.41	29	30.85	170	32.69	
> 5 times/week	20	3.26	1	1.06	19	3.65	0.255
Duration of tea consumption, y	10.99 ± 17.73		11.66 ± 20.31		10.87 ± 17.73		0.690

Bold type indicates statistical significance ($p < 0.05$).
Data presented are means (standard deviations) or number (%), as appropriate for the variable.

Subjects included in the analysis were younger ($p < 0.001$); more likely to be previously diagnosed with diabetes ($p = 0.018$); and with higher education levels ($p = 0.015$) and shorter duration of diabetes ($p = 0.031$). There was little difference between the two groups in the variables related to tea consumption, gender, occupation, individual monthly income, the status of smoking and alcohol consumption (all $p > 0.05$).
